# Biotechnological production of limonene in microorganisms

**DOI:** 10.1007/s00253-016-7337-7

**Published:** 2016-02-26

**Authors:** Esmer Jongedijk, Katarina Cankar, Markus Buchhaupt, Jens Schrader, Harro Bouwmeester, Jules Beekwilder

**Affiliations:** Laboratory of Plant Physiology, Wageningen University, Droevendaalsesteeg 1, Wageningen, 6708 PB The Netherlands; Plant Research International, PO Box 16, 6700 AA Wageningen, The Netherlands; DECHEMA Research Institute, Biochemical Engineering, Theodor Heuss-Allee 25, 60486 Frankfurt am Main, Germany

**Keywords:** Limonene, Biomaterial, Monoterpene, Microbial production, Metabolic engineering, Toxicity

## Abstract

This mini review describes novel, biotechnology-based, ways of producing the monoterpene limonene. Limonene is applied in relatively highly priced products, such as fragrances, and also has applications with lower value but large production volume, such as biomaterials. Limonene is currently produced as a side product from the citrus juice industry, but the availability and quality are fluctuating and may be insufficient for novel bulk applications. Therefore, complementary microbial production of limonene would be interesting. Since limonene can be derivatized to high-value compounds, microbial platforms also have a great potential beyond just producing limonene. In this review, we discuss the ins and outs of microbial limonene production in comparison with plant-based and chemical production. Achievements and specific challenges for microbial production of limonene are discussed, especially in the light of bulk applications such as biomaterials.

**Electronic supplementary material:**

The online version of this article (doi:10.1007/s00253-016-7337-7) contains supplementary material, which is available to authorized users.

## Introduction

Limonene is a well-known cyclic monoterpene. It is an olefin hydrocarbon (C_10_H_16_), which can occur in two optical forms. (+)-Limonene is one of the most important and widespread terpenes in the flavor and fragrance industry. Limonene (in both optical forms) has been found in more than 300 plant essential oils (DNP 2015) from very diverse species including orange, lemon, mint, and fir. Its biosynthesis has been well described in the plant kingdom. Limonene has been detected naturally in trace amounts in the headspace of microbes (Effmert et al. 2012; Heddergott et al. 2014; Hung et al. 2013); however, to our knowledge, no corresponding biosynthetic mechanism has been identified. By transformation with plant limonene synthases, microorganisms such as yeast and bacteria have been engineered to produce limonene. In this work, biotechnological production of limonene for application as commodity chemical is reviewed. Others have reviewed general aspects of production of terpenes in microbes and plants (Aharoni et al. 2006; Duetz et al. 2003; Kirby and Keasling 2009; Vickers et al. 2014; Wang et al. 2015). Recently, Lange (2015) reviewed the biosynthesis and biotechnology of limonene for flavor and fragrance applications.

New applications of limonene for fuel and biomaterials ask for large and stable production volumes. Metabolic engineering strategies, like overexpressing precursor pathway enzymes, have been applied for the purpose of increasing limonene titers, which are at the moment still far from the maximal theoretical yield. Crucial in such strategies is the overproduction of geranyl diphosphate (GPP), the direct precursor of limonene. New opportunities to increase yield will be discussed, including novel strategies for capturing the product from the microbial cultures and possibilities for relieving limonene toxicity. When successful, these optimization strategies could result in a role for limonene-based products in the bio-based economy.

## Applications and products from limonene

**Fig. 1:**
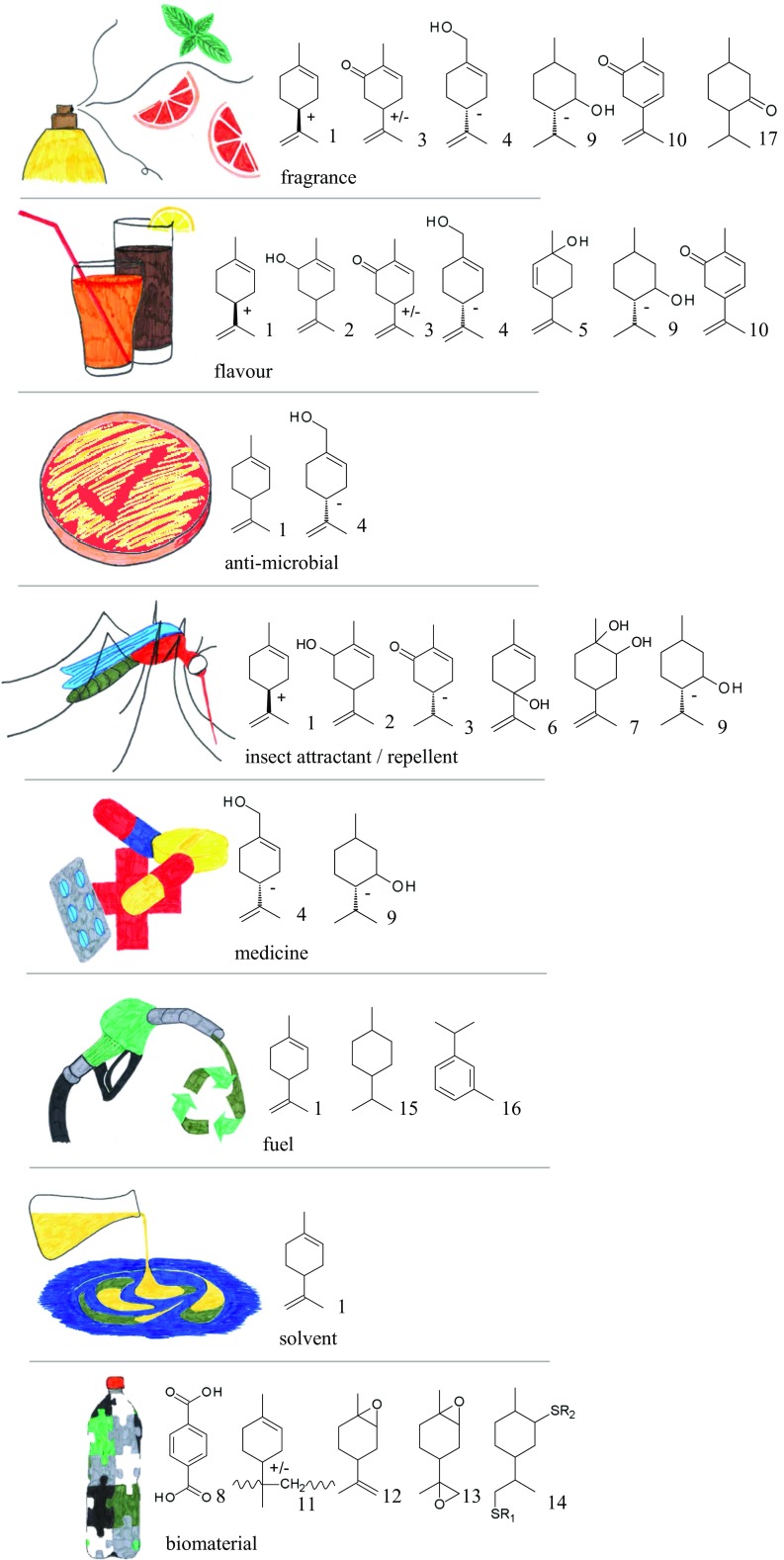
Applications of limonene and limonene-derived molecules made by plants (1, 2, 3, 4, 8, 9, 10), microbes (1, 2, 3, 4, 5, 6, 7, 8, 12), and/or chemical synthesis (1, 3, 11, 12, 13, 14, 15, 16, 17) (Duetz et al. 2003; Lerin et al. 2010; Duetz et al. 2001; Bowen 1975; Weldon et al. 2011, Tripathi et al. 2009, Lange 2015, Colonna 2011, Ciminno 1998, Ciriminna 2014, Firdaus 2011, Tracy 2009). *1* Limonene; *2* carveol; *3* carvone; *4* perillyl alcohol; *5* p-mentha-2,8-diene-1-ol; *6* p-mentha-1,8-dien-4-ol; *7* p-menth-8-ene-1,2-diol; *8* terephthalic acid; *9* menthol; *10* dehydrocarvone; *11* polylimonene; *12* limonene monoepoxide; *13* limonene di-epoxide; *14* product of thiol di-addition (R1 and R2 are thiol-side groups, e.g., 2-mercaptoethanol, methyl thioglycolate, or thioglycerol); *15* 1-isopropyl-4-methylcyclohexane; *16* m-cymene; *17* menthone; + and – indicate where a single enantiomer is used; +/− means either enantiomer can be used, but not as a mixture

Limonene has a wide variety of applications, which differ in volume, quality requirements, and price (Ciriminna et al. 2014) (Fig. 1). Traditionally, (+)-limonene is used as a flavoring compound in citrus-flavored products such as soft drinks and candy and as a fragrance ingredient in household cleaning products and perfumes (Duetz et al. 2003). As a flavor and fragrance ingredient, limonene has a relatively high price because of the quality requirements in this field. For this application, chirality is important. (+)-Limonene (also called R- or d-limonene) has a pleasant, orange-like odor whereas the (−)-form (also called S- or l-limonene) has a more harsh turpentine-like odor with lemon note (Friedman and Miller 1971). Limonene has minor applications in other products. For example, it is used as insecticide (Ciriminna et al. 2014) and is being investigated for medical applications due to its anti-microbial and anti-cancer properties (Inouye et al. 2001; Miller et al. 2010).

Potentially, limonene can also be used for larger-scale applications, for example, as an alternative to so-called benzene, toluene, ethylbenzene, and/or xylene (BTEX) solvents that are used in substantial volumes for oil and gas production (Fischer 2013). In addition, jet fuel replacements can be supplemented with limonene (Renninger et al. 2008). For solvent and fuel applications, large volumes of limonene at a low price would be required; however, exact numbers have, to our knowledge, not been reported.

The structure of limonene is very suitable for chemical modifications, due to the two available double bonds and possibility for hydroxylation (Wilbon et al. 2013). Modifications are important for many applications. Natural derivatives of limonene, mainly oxidized forms (Fig. 1), are used in particular for flavoring. For instance, the mint flavoring agent (−)-menthol is isolated from *Mentha* oil (Lange 2015). But, limonene is also suitable for (additional) chemical modifications (Fig. 1). For example, after complete hydrogenation, limonene can be added to diesel, to lower the cloud point and decrease its viscosity (Tracy et al. 2009). Modifications usually increase the price of the product; for example, limonene is sold at 9–10 $/kg, while (−)-menthol makes 15–40 $/kg (Lange 2015; Stuart Clark 1998).

Compared to the traditional use of limonene as flavor and fragrance ingredient, its application in the chemical industry has not received much attention in the scientific literature. However, biomaterials will be increasingly important to replace traditional, petrochemical-based materials (Langeveld et al. 2010). Several types of biomaterials can be made from limonene. A widely applied polymer of limonene, polylimonene (Piccolyte C115), is made from citrus oil (Cimmino et al. 1999). It is used as a resin in adhesives, as thermoplastics for the food packaging industry and electro-conductive parts, and as a masticatory agent in chewing gum. Terpene resins have suitable compositions for medical purposes such as drug delivery (Barros et al. 2007). Epoxidation of limonene yields limonene monoepoxide or di-epoxide (Fig. 1), which can subsequently be polymerized (Ciriminna et al. 2014). Limonene epoxide polymers are used in metal coatings, varnishes, and printing inks (Firdaus et al. 2011). Attaching two thiols to limonene (Fig. 1) facilitates polymerization to for, example, limonene/fatty-acid based polyesters (Firdaus et al. 2011). These are used as sealants and adhesives (Ciriminna et al. 2014). For many limonene-based biopolymers, chirality is important. Enantiopure limonene production from plants or microbes provides opportunities for chiral polymers with applications as chiral purification, nonlinear optics, or conducting materials (Firdaus et al. 2011). Limonene can also be converted to terephthalic acid (Fig. 1), which is used as building block for polyethylene terephthalate (PET) plastic (Colonna et al. 2011). PET is a widely used packaging material. The worldwide production of PET in 2009 was approximately 13 million tons (Colonna et al. 2011). Clearly, for commercial use of limonene in such biomaterials, affordable and reliable production at large volumes would be required.

## Current production of limonene

Limonene is available from various sources. Most limonene currently on the market is (+)-limonene, produced as a side product from the citrus juice industry. Citrus oil can contain 70–98 % of (+)-limonene (Sokovic et al. 2010; Tranchida et al. 2013). It is produced in more than 60,000 t/year (Lange 2015). Availability of citrus oil has been under pressure lately. Important citrus production areas in Brazil and the USA have been infested by the bacterial disease Huanglongbing (HLB), which has led to a drop in yield and a reduced area for citrus production (Hodges and Spreen 2012). Prices for citrus fruit, citrus oil, and limonene are therefore fluctuating and increasing. Currently, there are no successful disease management strategies for control of HLB, and therefore, availability of citrus-derived limonene is expected to continue to decrease. Besides that, part of the citrus-derived limonene is not food-grade, as it may contain significant amounts of pesticides (Nichkova et al. 2009), which limits the application in food and household products. Another source of limonene is turpentine, from which racemic limonene (referred to as dipentene) is produced at 450 t/year in the USA (Thorp 2010). Fully synthetic limonene can be made by Diels-Alder addition of two isoprene units. A process based on this addition has been described, which converts scrap tire rubber to limonene (Hanson et al. 1999). The scale at which fully synthetic limonene is produced is limited (Lange 2015). For limonene that needs to be food-grade or enantiopure, not all current sources are suitable.

Biotechnological production of limonene may complement current production systems. Microbial production would reduce dependence on the citrus industry and can convert raw materials like glucose or glycerol, which are available from a large variety of agricultural sources, to limonene. To avoid competing claims with food production, microbial biotechnology is developing ways to deploy biomass from non-food sources as a feedstock for microbial cultures (Rumbold et al. 2009). Microbially produced limonene and its derivatives may, in many cases, be labeled as “natural,” which has consequences for market prices (Serra et al. 2005). Microbial limonene synthesis is enantio-specific, which is necessary for applications in flavor and fragrance products and for chiral polymers. Moreover, oxidation by biocatalysts can be integrated in microbial production systems (Alonso-Gutierrez et al. 2013). The large volumes of limonene necessary for biobased solvents, fuel additives, and materials would make microbial production systems valuable on the longer term.

Costs of production of limonene in microbes have not been reported yet. For the related sesquiterpene farnesene, a jet fuel substitute, the costs for manufacturing in yeast have been reported to be as low as US$ 1.75 per liter (McCoy 2015). Clearly, if prices of microbially produced limonene drop to the same level, they reach the same order of magnitude as the current price for citrus-derived limonene. Farnesene yields of more than 100 g/L culture have been reported (Pray 2010). To our knowledge, the highest reported limonene titer so far was 1.35 g limonene per liter culture (Willrodt et al. 2014) and would need to increase still two orders of magnitude to reach the current price of plant-derived limonene.

## Biosynthesis of limonene

Optimization of microbial limonene production can be inspired by plants. Plants produce and store limonene in specialized structures. In citrus species, (+)-limonene is stored in secretory cavities in the peel of the fruit (Voo et al. 2012). These cavities are located in the outer, colored region of the peel, the flavedo. Biosynthetic genes for the production of limonene have been found to be highly expressed in epithelial cells that are lining the secretory cavities. The required enzymes were shown to be localized in organelles present in these cells, called leucoplasts (Turner and Lange 2015). Leucoplasts are plastids and differ from chloroplasts in that they lack photosynthetic machinery. In plant species of the *Lamiaceae* family, (−)-limonene and its derivatives accumulate in glandular trichomes, small structures on the surface of the leaves (Voirin and Bayet 1996). The enzymes involved in limonene biosynthesis and downstream oxidation are active in the secretory cells of these glandular trichomes (Turner et al. 1999; Turner and Croteau 2004). Limonene is stored in the subcuticular cavity of the trichome. The high concentrations of limonene found in the subcuticular cavities prove that trichomes should be considered as true cell factories (Lange and Turner 2013; Tissier 2012). Both in trichomes and in secretory cavities, limonene is stored outside the plant cells. If limonene concentrations in unspecialized plant cells become very high, the plant responds by emission of limonene into the air (Aharoni et al. 2005) or the glycosylation of limonene oxidation products (Fujita and Nakayama 1993; Lucker et al. 2001).

**Fig. 2:**
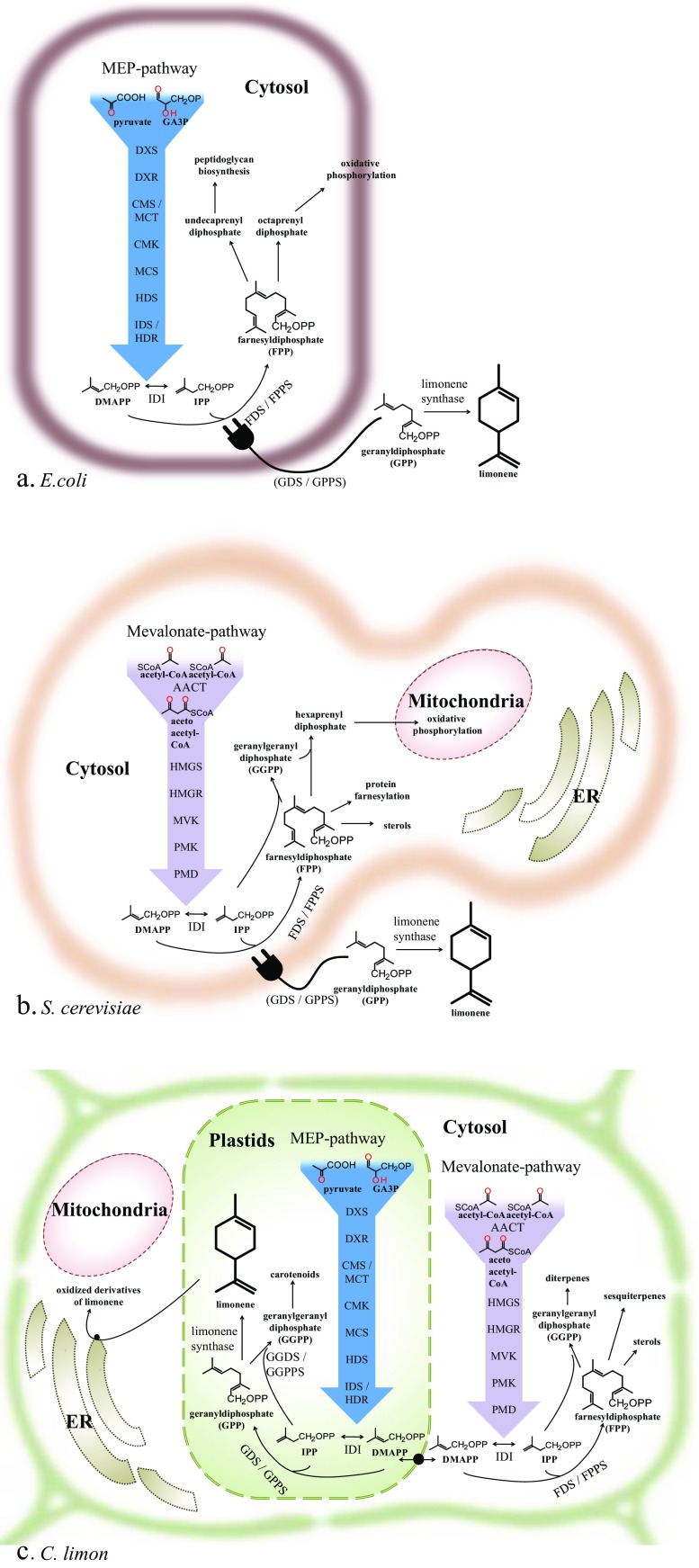
Terpene backbone biosynthesis in microorganisms and plants. *Plug* indicates plug-in of limonene biosynthesis in microbial hosts. *MEP* methylerythritol 4-phosphate, *ER* endoplasmic reticulum, *GA3P* d-glyceraldehyde 3-phosphate, *CoA* coenzyme A, *DXS* 1-deoxy-d-xylulose-5-phosphate synthase, *DXR* 1-deoxy-d-xylulose-5-phosphate reductoisomerase, *CMS/MCT* 4-diphosphocytidyl-2-C-methylerythritol synthase/2-C-methyl-d-erythritol-4-phosphate cytidylyltransferase, *CMK* 4-(cytidine-5′-diphospho)-2-C-methyl-d-erythritol kinase, *MCS* 2-C-ethyl-d-erythritol-2,4-cyclodiphosphate synthase, *HDS* 4-hydroxy-3-methylbut-2-enyl-diphosphate synthase, *IDS* isopentenyl diphosphate/dimethylallyl diphosphate synthase, *HDR* 4-hydroxy-3-methylbut-2-enyl-diphosphate reductase, *AACT* aceto acetyl-CoA thiolase, *HMGS* 3-hydroxy-3-methylglutaryl-CoA synthase, *HMGR* 3-hydroxy-3-methylglutaryl-CoA reductase, *MVK* mevalonate kinase, *PMK* phosphomevalonate kinase, *PMD* diphosphomevolonate decarboxylase, *IDI* isopentenyl diphosphate isomerase, *DMAPP* dimethylallyl diphosphate, *IPP* isopentenyl diphosphate (Banerjee and Sharkey 2014; Miziorko 2011; Rodriguez-Concepcion and Boronat 2002)

In plants, the biosynthesis of terpenes can proceed through two distinct isoprenoid-synthesizing pathways, which have been reviewed extensively (Banerjee and Sharkey 2014; Miziorko 2011; Rodriguez-Concepcion and Boronat 2002). Limonene is produced by limonene synthases from the substrate GPP (Fig. 2) (Lücker et al. 2002). GPP is a C_10_ compound that originates from the methylerythritol 4-phosphate (MEP) pathway. The MEP pathway produces the C_5_ units dimethylallyl diphosphate (DMAPP) and isopentenyl diphosphate (IPP) that are condensed to form GPP, facilitated by a prenyltransferase enzyme, GPP synthase. The two C_5_ units are also the building blocks for higher isoprenoids, for example, geranylgeranyl diphosphate (GGPP), the C_20_ building block for the carotenoid pathway and diterpenes. The MEP pathway operates in the plastids. A parallel pathway, the mevalonate pathway, operates in the cytosol (Fig. 2) and also delivers the same two C_5_ building blocks. This pathway is mostly used to supply the C_15_ substrate farnesyl diphosphate (FPP), for biosynthesis of sesquiterpenes and sterols.

### Limonene synthases

It is relevant for microbial production efficiency to use an appropriate limonene synthase. Synthases can differ in their product and enantiospecificity and performance in microorganisms. Synthases that convert GPP to limonene have been identified in 27 plant species from nine plant families (Table S1). Most limonene synthases produce almost exclusively limonene, but in some cases, limonene is one of several products (Lücker et al. 2002). Synthases often produce one predominant enantiomer of limonene, either (−) or (+). Remarkably, enantiospecificity can differ between limonene synthases from the same plant family. For example, within the *Lamiaceae* family, limonene synthase from *Perilla frutescens* makes predominantly (−)-limonene (Jongedijk et al. 2015), while limonene synthase from *Lavandula angustifolia* produces predominantly (+)-limonene (Landmann et al. 2007). Interestingly, a limonene synthase from the glandular trichomes of the wild tomato *Solanum habrochaites* has been described that deploys neryl diphosphate (NPP) as a substrate to make limonene, instead of GPP (Schilmiller et al. 2009). NPP is also a C_10_ diphosphate, but with a different stereochemistry than GPP, and is made by an NPP synthase (Kang et al. 2014). NPP-derived monoterpenes have also been found in soybean, indicating that NPP-dependent monoterpene biosynthesis may occur in more species (Zhang et al. 2013).

All known limonene synthases carry a plastid transit peptide, which mediates localization to the plastid, but is not present in the final, active form of the enzyme. Proper microbial expression of the limonene synthase requires removal of the transit peptide, which is not functional in microbes and may interfere with correct folding of the protein. Indeed, removal of the transit peptide improved limonene yields 4- to 8-fold in yeast (Jongedijk et al. 2015). In all the microbial production systems described below, the predicted transit peptide was removed.

## Producing limonene in microorganisms

Several choices have to be made when engineering a microbe for the production of limonene. This starts already with the choice of the microorganism, which may have consequences for the possibilities of feedstocks, possibilities for engineering, and system properties like solvent tolerance. The production of limonene in microorganisms can, in principle, be achieved by simply expressing a plant limonene synthase (Carter et al. 2003), but this has resulted in disappointing yields, likely due to the low availability of GPP in microorganisms. For an economically successful production of limonene in microbes, a metabolic engineering approach is required, directed at increasing the availability of GPP. Metabolic engineering choices that have been described, and remaining challenges, will be discussed in more detail.

### Microbial hosts used for limonene production

Naturally, microorganisms carry only one of the isoprenoid precursor pathways, either the MEP or the mevalonate pathway. In *Escherichia coli* and *Saccharomyces cerevisiae*, the two hosts commonly used for metabolic engineering of monoterpene production, only minor amounts of GPP precursor are available. Mostly, this GPP is a by-product from endogenous prenyltransferases, as a short-lived intermediate- to longer-chain isoprenoids (Burke and Croteau 2002) (Fig. 2). *E. coli* has a cytosolic MEP pathway, which normally functions to produce C_15_ FPP and higher polyprenyl diphosphates, which are used for biosynthesis of peptidoglycan, a cell wall component, and for production of ubiquinone for oxidative phosphorylation (Erhardt et al. 2014; Swiezewska and Danikiewicz 2005) (Table 1).

**Table 1:** Microbial strains engineered to produce limonene

Host	Engineering design	Limonene synthase origin, (+/−)-limonene, accession number	Maximal limonene yield per liter culture and recovery method	Reference
*E. coli* BLR (DE3) codon +	*•Abies grandis* tGPPS	*Mentha spicata*, (−), L13459	5 mg/L (−)-limonene; steam distillation	Carter et al. (2003)
*E. coli* DH1 ∆acrAB	*•*HMGS and tHMGR from *Staphylococcus aureus* •Codon optimized MVK, PMK, and PMD from *S. cerevisiae* •AACT and IDI from *E. coli* •tGPPS from *Abies grandis* •One plasmid containing the mevalonate pathway genes and one plasmid with tGPPS and tLS *•A. borkumensis* efflux pump (YP_692684)	*M. spicata*, (−), L13459, codon optimized	57 mg/L (−)-limonene; dodecane organic phase	Dunlop et al. (2011)
*E. coli* DH1	•HMGS and tHMGR from *Staphylococcus aureus* •Codon optimized MVK, PMK, and PMD from *S. cerevisiae* •AACT and IDI from *E. coli* •tGPPS from *Abies grandis* •One plasmid	*M. spicata*, (−), accession number not clear, codon optimized	430 mg/L (−)-limonene; dodecane organic phase	Alonso-Gutierrez et al. (2013)
*E. coli* BL21(DE3)	•HMGS and tHMGR, MVK, PMK, and PMD from *S. cerevisiae* •AACT and IDI from *E. coli* •tGPPS from *Abies grandis*/GPPS from *Streptomyces* sp. strain KO-3988	*M. spicata*, (−), L13459, codon optimized	1.35 g/L (−)-limonene; diisonoyl phthalate organic phase	Willrodt et al. (2014)
*Synechocystis* sp. PCC 6803	•DXS, IDI, and CrtE from *Synechocystis*	*Schizonepeta tenuifolia*, enantioselectivity not clear, AF282875	56 μg/L culture/day; gas stripping	Kiyota et al. (2014)
*Synechococcus* sp. PCC 7002	•Wild-type and Δ*glgC* background were compared	*M. spicata*, (−), Q40322, codon optimized	4 mg/L in wild-type background; dodecane organic phase	Davies et al. (2014)
*S. cerevisiae* AE9	•Yeast FPPS (ERG20 K197G) mutated to partly produce GPP	*Perilla frutescens*, (−), KM015220 and *Citrus limon*, (+), AF514287	0.49 mg/L (−)-limonene, 0.12 mg/L (+)-limonene; headspace trapping	Jongedijk et al. (2015)
*S. cerevisiae* EPY210C	•tHMGR from *S. cerevisiae* •upc2–1 transcription factor	*M. spicata*, (−), L13459 and *C. limon*, (+), AF514287, codon optimized	1.48 mg/L (−)-limonene dodecane organic phase	Behrendorff et al. (2013)

*GPPS* geranyl diphosphate synthase, *t* truncated, *LS* limonene synthase, *HMGS* 3-hydroxy-3-methylglutaryl-CoA synthase, *HMGR* 3-hydroxy-3-methylglutaryl-CoA reductase, *MVK* mevalonate kinase, *PMK* phosphomevalonate kinase, *PMD* diphosphomevolonate decarboxylase, *AACT* aceto acetyl-CoA thiolase, *IDI* isopentenyl diphosphate isomerase, *KO* knockout, *DXS* 1-deoxy-d-xylulose-5-phosphate synthase

While *E. coli* engineering has so far resulted in the highest yields of limonene, other microorganisms may offer advantages. *Saccharomyces* encodes a cytosolic mevalonate pathway (Fig. 2), which supplies FPP for biosynthesis of sterols (Takami et al. 2012) and for protein farnesylation (Dolence and Poulter 1995), and supplies GGPP for ubiquinone biosynthesis (Meganathan 2001). In yeast, fewer reports on limonene production are available and the titers reached are still lower than in *E. coli* (Table 1). However, yeast is more suitable for the co-expression of terpene synthases with subsequent oxidizing enzymes, which are often membrane-bound plant P450 enzymes with their corresponding NADPH-cytochrome P450 reductases (Gruchattka et al. 2013). Yeast has other advantages compared to *E. coli* in terms of tolerance to pH, osmotic pressure, and culture infections (Gruchattka et al. 2013).

Among the rare microbial species that do produce monoterpenes are *Streptomyces* species and cyanobacteria, many of which produce the monoterpene methylisoborneol (Giglio et al. 2011; Komatsu et al. 2008). Cyanobacteria are photosynthetic microorganisms, which are able to use CO_2_ and light as sources for limonene production (Davies et al. 2014). Using CO_2_ as a carbon source could, in principle, contribute to a highly sustainable production platform.

### Creating a GPP pool in microorganisms

A well-known strategy in the microbial engineering of terpene production is to overexpress mevalonate or MEP pathway enzymes with the terpene synthase. For example, in the cases of the microbial production of the sesquiterpenes trans-β-farnesene (a jet fuel substitute) and amorphadiene (a precursors of the anti-malarial artemisinin), this was achieved by overexpressing the mevalonate pathway together with the sesquiterpene synthases in an industrial yeast strain (George et al. 2015) (Martin et al. 2003).

For limonene production, this strategy has been successfully applied to *E. coli* by adding a mevalonate pathway (Alonso-Gutierrez et al. 2013; Dunlop et al. 2011; Willrodt et al. 2014). In *E. coli*, the level of limonene formation correlated with acetate formation, which was described as a side effect of the heterologous mevalonate pathway (Willrodt et al. 2014). In the cyanobacterium *Synechocystis* sp. PCC 6803, enzymes from the MEP pathway were added, resulting in modest improvements of limonene titers (Kiyota et al. 2014). This might have to do with the (unknown) product specificity of the prenyl diphosphate synthase crtE. An additional engineering strategy used to increase limonene production includes overexpression of a truncated version of 3-hydroxy-3-methylglutaryl-CoA reductase (tHMGR). HMGR is the key regulatory enzyme of the mevalonate pathway, and truncation by deletion of its N-terminus will overcome feedback inhibition of this pathway (Alonso-Gutierrez et al. 2013; Behrendorff et al. 2013; Dunlop et al. 2011; Willrodt et al. 2014).

One strategy to promote GPP availability is to express truncated versions of microbial GGPP or FPP synthases. These truncations can lead to enzymes that predominantly produce GPP (Narita et al. 1999). However, several disadvantages of this concept have been reported, for example, a negative influence on reaction kinetics (Reiling et al. 2004) or formation of by-products caused by solvolysis of GPP in the enzymatic pocket (Fischer et al. 2011; Jongedijk et al. 2015).

As an alternative to mutant enzymes, a true GPP synthase can be introduced, which has so far only been described for plants and for *Streptomyces* (Willrodt et al. 2014). Many plant GPP synthases have been reported; however, not all of them are equally suited for microbial metabolic engineering. Most convenient for this purpose are homodimeric synthases, such as those from *Arabidopsis* (Bouvier et al. 2000) and *Abies* (Burke and Croteau 2002). Also, heterodimeric GPP synthases have been described, for example, from peppermint *Mentha* × *piperita* (Chang et al. 2010), but balanced biosynthesis of the two subunit genes is still challenging. Importantly, most GPP synthases appear to mediate also FPP and/or GGPP biosynthesis in vitro (Burke and Croteau 2002). Such enzymes may play a dual role in the biosynthesis of monoterpenes and diterpenes as, for example, has been reported for tomato GPP synthase (van Schie et al. 2007). For reasons of efficiency, a synthase that produces exclusively GPP would be advantageous for a monoterpene biosynthetic microorganism. Therefore, many reports on limonene biosynthesis in microbes have used the *Abies grandis* GPP synthase (Alonso-Gutierrez et al. 2013; Carter et al. 2003; Willrodt et al. 2014), which produces predominantly GPP (Burke and Croteau 2002). Tuning of the expression and solubility of GPP synthases in the microorganism appeared to have strong effects on productivity (Alonso-Gutierrez et al. 2013), demonstrating the importance of this enzyme for a successful limonene production system.

## Capturing produced limonene from fermentation systems

In microbial production systems, downstream processing usually constitutes a considerable part of the costs. The recovery of limonene from fermentation systems needs attention due to its high volatility and anti-microbial nature (Jongedijk et al. 2015; Leng et al. 2013). Several capturing methods have been reported on lab scale, including culture extraction, solvent overlay, solid-phase micro-extraction (SPME), an adsorbent polydimethylsiloxane bar (Twister^®^), continuous headspace trapping, and gas stripping to a cold trap (Behrendorff et al. 2013; Ignea et al. 2014; Jongedijk et al. 2015; Kiyota et al. 2014; Vararu et al. 2015). Not all of these methods are suitable for industrial-scale recovery of limonene. Most suitable for also larger production scale are the ones in which limonene is continuously removed during culturing, for example, by a two-phase system or by headspace removal. These strategies prevent product inhibition and toxicity effects and avoid evaporative loss of the produced limonene. For example, Brennan et al. (2012) showed that using an overlay of dibutyl phthalate could increase the minimal inhibitory concentration of limonene to yeast by up to 702-fold and thus alleviate its toxicity. In cyanobacteria, an overlay of dodecane enhanced limonene recovery (Davies et al. 2014). In the absence of a solvent layer, it is important to continuously trap limonene from the culture headspace, in order to minimize not only losses but also possible toxic effects. Jongedijk et al. (2015) showed that continuous capturing of limonene from a yeast culture headspace results in an 8-fold higher limonene yield, compared to extraction by a solvent overlay.

While volatility and toxicity limit the choice of methods for limonene recovery from microbial production systems, this choice may also depend on the subsequent application of limonene. If limonene needs to be obtained as a pure essential oil, for example, for fragrance application, it might be preferable to use a solvent-free system. If limonene is to be used as an additive to a fuel or solvent, it might be preferable to use this matrix already as overlay during culturing (Brennan et al. 2012).

## Toxicity and solvent tolerance

High titers of limonene are, in principle, incompatible with the fact that limonene exerts strong toxic effects on cells (Andrews et al. 1980; Uribe and Pena 1990). Limonene is highly lipophilic (Griffin et al. 1999), which causes accumulation of limonene in biological membranes. Disruption of the cell membrane integrity as well as inhibition of essential membrane functions is the mechanism underlying the general toxicity of solvents such as monoterpenes (Sikkema et al. 1994). The mechanisms of microbial solvent tolerance, especially in *Pseudomonas* species, have been studied in detail (reviewed, e.g., by Segura et al. 2012; Ramos et al. 2015), although mostly with regard to organic solvents other than monoterpenes. Changes in the cellular energy homeostasis, alterations of cell membrane structure, increased formation of chaperones, induction of proteins dealing with reactive oxygen species, and activation of efflux pump systems are the main cellular responses observable after exposure of microbes to organic solvents. Especially, *Pseudomonas putida* shows extraordinarily high tolerance toward many organic molecules (Ramos et al. 2015) and has been demonstrated to serve as a suitable microorganism for processes with high amounts of externally added limonene (Mirata et al. 2009).

In microbial limonene production, a high limonene export rate is required to avoid intracellular accumulation. A screen for efflux pumps that increase tolerance of *E. coli* toward added limonene identified the AcrAB pump of *E. coli* as well as an efflux pump of *Alcanivorax borkumensis* (Dunlop et al. 2011). Expression of the latter in an *E. coli* strain producing non-growth inhibiting amounts of limonene resulted in a 30 % higher product concentration, probably due to reduced feedback inhibition of the limonene synthesis pathway. Tolerance of *S. cerevisiae* toward limonene and other monoterpenes could be enhanced by expression of a fungal efflux pump that was demonstrated to be involved in monoterpene resistance of the bark beetle-associated pine tree pathogen *Grosmannia clavigera* (Wang et al. 2013). Recently, evolutionary engineering of *S. cerevisiae* demonstrated a truncation of a tricalbin protein with a probable function in cell wall integrity regulation to confer a drastic improvement in tolerance toward limonene and other monoterpenes (Brennan et al. 2015).

A recent report (Chubukov et al. 2015) may reveal why externally added limonene at a concentration of about 0.025 % (*v*/*v*) completely inhibits growth of *E. coli*, whereas α-pinene is tolerated at much higher concentrations (Dunlop et al. 2011), and more than 0.04 % limonene (*v*/*v*) can be produced de novo from glucose by *E. coli* (Alonso-Gutierrez et al. 2013). Chubukov and colleagues identified limonene hydroperoxides as the main toxic compounds present in externally added limonene and could furthermore demonstrate that a single amino acid change in the alkyl hydroperoxidase AhpC alleviates this toxicity. An *E. coli* strain expressing this variant, AhpCL177Q, which is probably able to reduce the limonene hydroperoxides to less toxic monoterpene alcohols, still displayed more than 50 % of its maximal specific growth rate in a culture containing 10 % limonene (*v*/*v*) (Chubukov et al. 2015). The authors also demonstrated that addition of 2 % non-oxidized (anaerobically stored) limonene (*v*/*v*) did not lead to a clear reduction of the *E. coli* growth rate. When considering that limonene hydroperoxides are rapidly formed from limonene if stored under an oxygen-containing atmosphere (Karlberg et al. 1994), interpretation of most of the toxicity data from the publications described herein is difficult due to the lack of knowledge about the hydroperoxide content of the limonene used. Moreover, some of the efflux pumps might show transport activity toward limonene hydroperoxide, complicating direct comparisons between different proteins. It was nevertheless demonstrated that the AcrAB efflux pump is essential for the high resistance of the AhpCL177Q-expressing *E. coli* strain toward the limonene/limonene-hydroperoxide mixture (Chubukov et al. 2015). Although the limonene hydroperoxide issue might have strong impact on limonene biotransformation processes using growing cells, the authors furthermore stated that in limonene production processes, the toxicity of limonene hydroperoxides will only be relevant in long-lasting fermentations. Clearly, the control of cellular export and catabolism of limonene is important for reaching high limonene productivity in microbial systems.

## Conclusions

Limonene, which is now mostly used as a fragrance, has a wide variety of potential applications as a bulk material. A stable and scalable source of limonene is needed for production of biomaterials, solvents, and fuels. Microbial production could meet that demand but still needs significant engineering efforts. As yet, titers produced by various microbial systems would need to be improved at least two orders of magnitude for a price competitive with plant-derived limonene. The difference in productivity between plants and microbes may be explained by the adaptations of plant cells for limonene production. These include the natural presence of the precursor geranyl diphosphate (GPP) in specialized compartments and the ways that plants protect their cells from toxic effects by modification and/or extracellular storage of limonene. However, several research groups successfully engineered a GPP pool in microbes, using engineered microbial enzymes or plant GPP synthases. Microbes can suffer from the presence of limonene due to its anti-microbial properties. However, promising results have been reported to alleviate toxicity of limonene for microorganisms by capturing the product from the culture and increasing solvent tolerance. Further studies to increase the GPP pool, and alleviating toxicity effects of limonene on the used host, would make it possible to maximally exploit microorganisms to produce limonene for new bulk applications, such as solvents and biomaterials.

## Electronic Supplementary Material

ESM 1 (PDF 18 kb)
