# Supplementary material for: Biotechnological production of limonene in microorganisms
Source: Appl Microbiol Biotechnol. 2016 Feb 26;100:2927–38. doi: 10.1007/s00253-016-7337-7 (PMC4786606; doi:10.1007/s00253-016-7337-7)
Supplement: Supplementary file 1 — (PDF 18 kb) [file 253_2016_7337_MOESM1_ESM.pdf]

Supplemental Table S1

“Biotechnological production of limonene in microorganisms”

Applied Microbiology and Biotechnology

Esmer Jongedijk, Katarina Cankar, Markus Buchhaupt, Jens Schrader, Harro Bouwmeester and Jules Beekwilder*

*Correspondence to: J. Beekwilder. Plant Research International, PO Box 16, 6700 AA, Wageningen, The Netherlands. E-mail: jules.beekwilder@wur.nl, tel: +31 317 480979, fax: +31 317 418094

Table S1 Plant limonene synthases

**Plant species**	**GenBank accession nos.**	**Plant family**	**Stereo isomer[a]**	**Reference**	**Used in microbes (reference)**
Eleutherococcus trifoliatus	KJ126717	*Araliaceae*		Huang KF, Lee YR, Tseng YH, Wang SY, Chu FH (2015) Holzforschung 69(2):163-171	
Cannabis sativa	DQ839404	*Cannabaceae*	(-)	Guennewich N, Page JE, Kutchan TM (2007) Nat Prod Commun 2:223-232	
Arabidopsis thaliana	At3g25810	*Cruciferae*		Chen F, Tholl D, D'Auriaa JC, Farooqb A, Pichersky E, Gershenzon J (2003) Plant Cell 15(2):481-94.	
Oryza sativa	Os04g27340	*Graminae*		Lee GW, Lee S, Chung MS, Jeong YS, Chung BY (2015) Protoplasma 252(4):997-1007	
Mentha arvensis	JX555963	*Lamiaceae*		Wang H, Yu X, Li W, Liang C, Liu Y (2013) Gene 524:246-252	
Mentha cardiaca	JX555964-5	*Lamiaceae*		Wang H, Yu X, Li W, Liang C, Liu Y (2013) Gene 524:246-252	
Mentha haplocalyx	JX555966-9	*Lamiaceae*		Wang H, Yu X, Li W, Liang C, Liu Y (2013) Gene 524:246-252	
Mentha rotundifolia	JX555970-1	*Lamiaceae*		Wang H, Yu X, Li W, Liang C, Liu Y (2013) Gene 524:246-252	
Mentha spicata	JX555972-5; JN587785; AR036109-15; L13459; Q40322	*Lamiaceae*		Wang H, Yu X, Li W, Liang C, Liu Y (2013) Gene 524:246-252; Curto MA, Puppo P, Ferreira D, Nogueira M, Meimberg H (2012) Mol. Phylogenet. Evol. 63(3):758-767; Croteau RB, Colby SM (199) US patent 5871988 A; Colby SM, Alonso WR, Katahira EJ, McGarvey DJ, Croteau R (1993) J Biol Chem 268(31):23016-23024	*Escherichia coli* (Carter OA, Peters RJ, Croteau R (2003) Phytochem 64:425-433; Alonso-Gutierrez J, Chan R, Batth TS, Adams PD, Keasling JD, Petzold CJ, Lee TS (2013) Metab eng 19:33-41; Willrodt C, David C, Cornelissen S, Buhler B, Julsing MK, Schmid A (2014) Biotech J 9:1000-1012; Dunlop MJ, Dossani Z, Szmidt H, Chu HC, Lee TS, Keasling JD, Hadi MZ, Mukhopadyay A (2011) Mol Syst Biol 7:487)<br>*Saccharomyces cerevisiae* (Behrendorff JB, Vickers CE, Chrysanthopoulos P, Nielsen LK (2013) Microb cell fact 12:76)<br>*Synechococcus* sp. PCC 7002 (Davies FK, Work VH, Beliaev AS, Posewitz MC (2014) Front Bioeng Biotech 2:21)

Mentha canadensis	JX555976; JX555966-9	*Lamiaceae*		Wang H, Yu X, Li W, Liang C, Liu Y (2013) Gene 524:246-252	
Mentha suaveolens	JX555970-1	*Lamiaceae*		Wang H, Yu X, Li W, Liang C, Liu Y (2013) Gene 524:246-252	
Mentha gracilis	JX555964-5	*Lamiaceae*		Wang H, Yu X, Li W, Liang C, Liu Y (2013) Gene 524:246-252	
Perilla frutescens	KM015220; D49368	*Lamiaceae*	(-)	Jongedijk E, Cankar K, Ranzijn J, van der Krol S, Bouwmeester H, Beekwilder J Yeast 32(1):159-171; Yuba A, Yazaki K, Tabata M, Honda G, Croteau R (1996) Arch Biochem Biophys 332(2):280-287	*Saccharomyces cerevisiae* (Jongedijk E, Cankar K, Ranzijn J, van der Krol S, Bouwmeester H, Beekwilder J Yeast 32(1):159-171)
Perilla citriodora	AF233894	*Lamiaceae*		Ito M, Kiuchi F, Yang LL, Honda G (2000) Biol Pharm Bull 23(3):359-362	
Lavandula angustifolia	DQ263740	*Lamiaceae*	mainly (+)	Landmann C, Schwab W (2007) Arch Biochem Biophys 465(2):417-429	
Schizonepeta tenuifolia	AF282875	*Lamiaceae*		Maruyama T, Ito M, Kiuchi F, Honda G (2001) Biol Pharm Bull 24(4):373-377	*Synechocystis* sp. PCC 6803 (Kiyota et al., 2014 J Biotechnol 185; 1–7)
Micromeria lanata	JN587786	*Lamiaceae*		Curto MA, Puppo P, Ferreira D, Nogueira M, Meimberg H (2012) Mol. Phylogenet Evol 63(3):758-767	
Agastache rugosa	AY055214	*Lamiaceae*		Maruyama T, Saiki D, Ito M, Honda G (2002) Biol Pharm Bull 25:5, 661-665	
Picea sitchensis	DQ195275	*Pinaceae*		Byun-McKay A, Godard KA, Toudefallah M, Martin DM, Alfaro R, King J, Bohlmann K, Plant AL (2006) Plant Physiol 140(3):1009-1021	
Picea abies	AY473624	*Pinaceae*	mainly (-)	Martin DM, Faeldt J, Bohlmann J (2004) Plant Physiol 135(4):1908-1927	
Abies grandis	AF006193; AF326518	*Pinaceae*	(-)	Bohlmann J, Steele CJ, Croteau R (1997) J Biol Chem 272(35):21784-21792; Trapp SC, Croteau RB (2001) Genetics 158(2):811-832	
Coffea arabica	HE985292	*Rubiaceae*		Del Terra L, Lonzarich V, Asquini E, Navarini L, Graziosi G, Suggi Liverani F, Pallavicini A (2013) Phytochemistry 89:6-14	
Citrus jambhiri	AB266584	*Rutaceae*		Yamasaki Y, Akimitsu K 2007, J Plant Physiol 164(11):1436-1448	
Citrus unshiu	AB110636; AB110637	*Rutaceae*		Shimada T, Endo T, Fujii H, Hara M, Omura M (2004) Plant Sci. 166:49–58;	

				Shimada T, Endo T, Fujii H, Omura M 2005, Sci Hortic 105:507–512	
Citrus limon	AF514289; AF514287	*Rutaceae*	(+)	Lücker J, El Tamer MK, Schwab W, Verstappen FWA, van der Plas LHW, Bouwmeester HJ, Verhoeven HA (2002) Eur J Biochem 269:3160-3171	*Saccharomyces cerevisiae* (Jongedijk E, Cankar K, Ranzijn J, van der Krol S, Bouwmeester H, Beekwilder J Yeast 32(1):159-171; Behrendorff JB, Vickers CE, Chrysanthopoulos P, Nielsen LK (2013) Microb cell fact 12:76)
Solanum habrochaites	JN990693; NM_001247700	*Solanaceae*		Gonzales-Vigil E, Hufnagel DE, Kim J, Last RL Barry CS (2012) Plant J. 71(6): 921-935; Schilmiller AL, Schauvinhold I, Larson M, Xu R, Charbonneau AL, Schmidt A, Wilkerson C, Last R, Pichersky E (2009) P Natl Acad Sci USA 106:10865-10870	
Solanum lycopersicum	NM_001308378	*Solanaceae*		Falara V, Akhtar TA, Nguyen TT, Spyropoulou EA, Bleeker PM, Schauvinhold I, Matsuba Y, Bonini ME, Schilmiller AL, Last RL, Schuurink RC, Pichersky E (2011) Plant Physiol 157:2, 770-789	

[a]Stereo chemistry confirmed by experimental data
